# Pien Tze Huang Alleviates Relapsing-Remitting Experimental Autoimmune Encephalomyelitis Mice by Regulating Th1 and Th17 Cells

**DOI:** 10.3389/fphar.2018.01237

**Published:** 2018-10-31

**Authors:** Xuemei Qiu, Qingqing Guo, Xue Liu, Hui Luo, Danping Fan, Yongqi Deng, Hua Cui, Cheng Lu, Ge Zhang, Xiaojuan He, Aiping Lu

**Affiliations:** ^1^Institute of Basic Research in Clinical Medicine, China Academy of Chinese Medical Sciences, Beijing, China; ^2^School of Life Science and Engineering, Southwest Jiaotong University, Chengdu, China; ^3^Law Sau Fai Institute for Advancing Translational Medicine in Bone & Joint Diseases, Hong Kong Baptist University, Hong Kong, China; ^4^E-Institute of Internal Medicine of TCM, Shanghai University of Traditional Chinese Medicine, Shanghai, China

**Keywords:** experimental autoimmune encephalomyelitis, multiple sclerosis, Pien Tze Huang, Th1 cells, Th17 cells

## Abstract

Multiple sclerosis (MS) is an autoimmune disease of the central nervous system (CNS), characterized by infiltrating inflammatory cells and demyelinating lesions, and T helper (Th) cells play critical roles in the pathogenesis of MS. There is still lack of effective treatments currently. Pien Tze Huang (PZH), a traditional Chinese medicine formula, has been proved to have anti-inflammatory, neuroprotective, and immunoregulatory effects. However, whether PZH can be used to treat MS is still obscure. This study aimed to investigate the possible therapeutic effect and the underlying action mechanism of PZH in relapsing-remitting experimental autoimmune encephalomyelitis (RR-EAE) mice. Female SJL/J mice were immunized with myelin proteolipid protein 139–151 (PLP_139−151_) and pertussis toxin to establish RR-EAE model. Mice were then randomly divided into normal group, model group, PZH group and positive control group (fingolimod, FTY-720), and drugs were orally administered for 60 days from the day 10 after immunization. Sera of mice were collected for ELISA detection. Tissues of CNS were harvested for hematoxylin-eosin (H-E) and luxol fast blue (LFB) staining. Furthermore, Th1, Th17 cells and their related cytokines in the CNS were detected by flow cytometry and quantitative real-time PCR, respectively. Proteins involved in STAT and NF-κB signaling pathways were detected by western blot. The results showed that PZH-treated mice displayed mild or moderate clinical symptoms compared with untreated EAE mice that exhibited severe clinical symptoms. PZH remarkably reduced inflammatory cell infiltration and myelin damage in the CNS of EAE mice. It markedly down-regulated the levels of IFN-γ and IL-17A in sera of EAE mice. Moreover, PZH could reduce the percentages of Th1 and Th17 cells. It also suppressed the production of transcription factors ROR-γt and T-bet as well as the mRNA levels of their downstream pro-inflammatory cytokines, such as IFN-γ and IL-17A. Furthermore, PZH could inhibit the phosphorylation of some key proteins in the STAT and NF-κB signaling pathways. In conclusion, the study demonstrated that PZH had a therapeutic effect on RR-EAE mice, which was associated with the modulation effect on Th1 and Th17 cells.

## Introduction

Multiple sclerosis (MS) is the most common immune-mediated demyelinating disease of central nervous system (CNS) leading to damage of the myelin sheath and axons/neurons followed by neurological symptoms. Approximately 85% of MS patients present with a relapsing-remitting phenotype. Infiltration of myelin-reactive CD4^+^ T helper (Th) cells and release of pro-inflammatory cytokines lead to inflammatory infiltrates, demyelination, and axonal damage in the CNS as the major pathological finding in the MS (Thone and Linker, 2016). CD4^+^ T subsets involved in MS pathogenesis include Th1 cells, which primarily secrete pro-inflammatory cytokines interferon (IFN)-γ, and Th17 cells, which mainly produce interleukin (IL)-17A (Marino and Cosentino, 2016).

Currently, approved therapies for MS either have limited efficacy or pose significant safety concerns, leading to the development and investigation of new drugs (Riera et al., 2016). Traditional Chinese medicines (TCM) have recently received great interest since they have long been used to treat various inflammation-related diseases and have relatively few side-effects (Li et al., 2017; Zhu et al., 2017).

Pien Tze Huang (PZH), a Chinese medicine formula mainly consisting of musk (now replaced by artificial musk), calculus bovis, snake's gall, and *Panax notoginseng* (Burkill) F.H.Chen (Tianqi or Sanqi), has been found to possess anti-inflammatory, immunomodulation and neuro-protective effects (Lee et al., 2002; Lü et al., 2009; Zhang et al., 2010). It has been widely used in various inflammatory diseases in clinic. Experimental data displayed that PZH could inhibit the expression of proinflammatory cytokines, like IL-1β, IL-6, TNF-α, and so on. Also, PZH had the regulation effect on NF-κB which was closely related to the expression of inflammatory factors (Lee et al., 2002). Furthermore, PZH treatment could inhibit the phosphorylation level of STAT3, which was important in the development of Th17 cells and has a vital role in the pathogenesis of MS/EAE (Zhuang et al., 2012). In addition, the main components in PZH, such as Sanqi, has been proved to exert good effect on MS/EAE (Beamer and Shepherd, 2012; Zhu et al., 2014; Lee et al., 2016). Based on all these investigations, the effect of PZH on MS was firstly observed in our previous study by exploiting an acute EAE rat model and PZH showed a therapeutic effect on the EAE rats (Qiu et al., 2018). However, considering the limitation of the acute EAE model and the fact of relapsing-remitting phenotype being the dominant form in MS patients, PZH's effect on MS need more evidence from different EAE models.

Therefore, in order to further investigate the potential therapeutic effects and underlying mechanism of PZH in MS, relapsing-remitting experimental autoimmune encephalomyelitis (RR-EAE) mice model was used in the present study. Previous studies have shown that SJL/J mice immunized by synthetic myelin PLP_139−151_ which was an immunodominant epitope of myelin showed several relapses and remissions after an initial attack. The relapsing-remitting EAE in SJL/J mice involves several immune cells, including CD8, CD4, and Th17 cells and can be applied to study neuroinflammation and immune system activation in relapsing-remitting MS and test therapeutical agents (Procaccini et al., 2015).

## Materials and methods

### Drugs

PZH was obtained and authenticated from Zhangzhou Pientzehuang Pharmaceutical Co., Ltd., (Zhangzhou, China; FDA approval no. Z35020243). Fingolimod (FTY-720) was purchased from MedChemexpress (MCE, Monmouth Junction, NJ, USA).

### HPLC-MS/TOF study of PTH

PZH sample (1.0 g) was extracted with 50 ml 70% ethanol for 2 h by using soxlet extraction method. After filteration and evaporation, the remaining residue was dissolved in 10 ml 70% ethanol. The sample was then filtered by a 0.45 μm filter and determined by Agilent 1,200 HPLC apparatus (Agilent Technologies Inc., Palo Alto, USA). HPLC separation was performed on an Agilent Zorbax SB-C18 column (4.6 × 250 mm, 5 μm) using a mobile phase of acetonitrile and water at a flow rate of 1.0 ml/min, the column temperature was set as 25°C, and the detection wavelength was 203 nm. The gradient program (MeCN:H2O, v/v) were 22:78 (*t* = 0 min), 22:78 (*t* = 40 min), 30:70 (*t* = 50 min), 55:45 (*t* = 80 min), 55:45 (*t* = 95 min), 90:10 (*t* = 115 min), 90:10 (*t* = 135 min). The MS measurements were carried out by using the Agilent 6420 Accurate-Mass TOF LC/MS (Agilent Technologies, Santa Clara, CA, USA) under the following conditions at Nebulizer-35 psi, Dry Gas-9.0 L/min, Dry Temp-325°C.

### Animals

Forty female SJL/J mice, 6-8 weeks of age with a mean weight of 18~20 g were purchased from Beijing Vital River Laboratories (Beijing, China) [certification NO. SCXK (JING) 2012-0001]. Mice were housed five per cage in an appropriate environment with an air-filtering system. They were allowed to acclimatize themselves for 1 week prior to the initiation of experiment. All experimental procedures were approved by the Research Ethics Committee of China Academy of Chinese Medical Sciences and Hong Kong Baptist University (No.EACUC-17-0052, No. HASC/16-17/0045, respectively).

### EAE induction

For establishing relapsing-remitting EAE model, female SJL/J mice were immunized subcutaneously with 0.2 mL of emulsion containing 150 μg of myelin proteolipid protein139-151 (PLP_139−151_, HSLGKWL0GHPDKF; HPLC-purity >98%) (ChinaPeptides, Shanghai, China) dissolved in 0.1 mL sterile phosphate buffered saline (PBS) and 0.1 mL complete Freund's adjuvant (CFA) (Chondrex, Redmond, WA, USA) supplemented with 4 mg/mL Mycobacterium tuberculosis (Sands et al., 2014; Rovituso et al., 2016). These injections were distributed over two spots upper abdomen with 0.1 mL emulsion per site. Each mouse received an additional 200 ng pertussis toxin (PTX) (R&D Systems, MN, USA) diluted in 200 μL PBS by intraperitoneal injection on day 0 and day 2 post-immunization (p.i.). Clinical scores were calculated blindly by two researchers daily according to a 0–5 scale as follows (Kono et al., 1988): 1 = flaccid tail; 2 = moderate hind or front leg weakness; 3 = severe hind or front leg weakness; 4 = complete paralysis of limb; 5 = death.

### Grouping and treatment

PZH solutions were prepared prior to use by dissolving PZH powder in double distilled water to the final concentration. Mice were randomly divided into four groups with 10 mice per group: normal group, model group, PZH group (0.234 g/kg/d, equal to adult dose) and positive control group (fingolimod, FTY-720, 1 mg/kg/d) (Huggins and Sergott, 2011; Mehling et al., 2011; Sanford, 2014). The administrations of drugs started from day 10 after immunization (disease onset) and lasted for 60 days. PZH and FTY-720 were oral administered in a volume of 0.1 mL/10 g. The mice in normal group and model group were administered with the same volume of double distilled water.

### ELISA

Mice were sacrificed at day 70 after immunization and the sera were collected. The levels of IFN-γ and IL-17A in sera were detected with ELISA kits (eBioscience, San Diego, CA, USA) according to the manufacturer's protocol.

### Flow cytometry

Mice were anesthetized and perfused with cold PBS. The CNS tissues and spleens were collected and the mononuclear cells (MNCs) were isolated following published protocols (Rothhammer et al., 2011; Hammer et al., 2016). Simply, CNS tissues were digested in dulbecco's modified eagle medium (DMEM) supplemented with 2.5 mg/mL collagenase D (Roche Diagnostics, Indianapolis, IN) and 10 mg DNAseI (Sigma-Aldrich, St. Louis, MO) at 37°C for 30 min. A single cell suspension was prepared by passing the tissue through a 70 μm cell strainer and fresh Percoll (Sigma-Aldrich, St. Louis, MO) gradient (30/70%) centrifugation. MNCs from the interface between 70 and 30% layers were removed, washed, and re-suspended in culture medium for further analysis. Splenocytes were isolated from the spleen of mice after being homogenated and past the 70 μm cell strainer. Viable cells were counted in 0.4% Trypanblue. Then, cells (1 × 10^6^) were stimulated for 4 h at 37°C and 5% CO_2_ incubator in flow tube with 2 μL cell activation mixture containing PMA, ionomycin and brefeldin A from the Leukocyte Activation Cocktail, with BD GolgiPlug^TM^ (BD Biosciences, San Jose, CA). The detection of Th1 and Th17 cells were carried out using a Mouse Th1/Th17 Phenotyping kit (BD Biosciences, San Jose, CA) according to the manufacturer's instructions. After fixing and permeabilizing, cells were sorted by a BD Accuri™ C6 Flow Cytometer (BD Biosciences, San Jose, CA) and the data were analyzed with BD accuriCFlow Plus (BD Biosciences, San Jose, CA).

### Histopathology

All the mice were anesthetized and sacrificed at day 70. The brain and the spinal cord was collected and fixed in 10% neutral formalin for 48 h. Then the brain and the lumbar segments of spinal cord were treated with ethanol and xylene, and embedded in paraffin to obtain 6 μm-thick sections. And then the sections were stained by hematoxilin-eosin (H-E) for pathological observation and inflammation evaluation and Luxol fast blue (LFB) for demyelination assessment. An average of 8-10 sections of each brain and lumbar cord from each of five mice per group were scored as the following standard for evaluation (Kan et al., 2015; Belloli et al., 2018). For inflammation: 0 = no inflammatory cells; 1 = a few scattered inflammatory cells; 2 = organization of inflammatory infiltrates around blood vessels; 3 = extensive perivascular cuffing with extension into parenchyma. For demyelination: 0 = none; 1 = rare foci; 2 = a few areas of demyelination; and 3 = large (confluent) areas of demyelination.

### Western blot

The levels of phosphorylated (p)-STAT1 (rabbit polyclonal antibody, dilution 1:1,000, CST, Boston, USA), p-STAT3 (rabbit monoclonal antibody, dilution 1:1,000, CST, Boston, USA), p-STAT4 (rabbit polyclonal antibody, dilution 1:500, Abcam, Cambridge, UK), NF-κB P65 (rabbit polyclonal antibody, dilution 1:4,000, Abcam, Cambridge, UK), p-P65 (rabbit polyclonal antibody, dilution 1:500, Abcam, Cambridge, UK) in the brain of mice were detected by western blot. Firstly, the brains of mice were collected and snap frozen at −80°C immediately. Next, the brain homogenates were prepared in lysis buffer (P0013) consisting of 1 nM PMSF and phosphatase inhibitor. Proteins were denatured and equal amounts of proteins were electrophoresed in 8% bis-Tris/polyacrylamide gels (P0012A; Beyotime, shanghai, China) and transferred to PVDF membranes with 0.45 μm pores (Millipore, Boston, MA, USA). The membranes were blocked for 2 h in blocking solution (TBS containing 5% nonfat dry milk and 0.1% Tween 20) and incubated overnight at 4°C with primary antibody diluted in blocking solution, followed by incubation with horseradish peroxidase-conjugated secondary antibody at room temperature for another 2 h. Last, the immunoreactivity was detected with enhanced chemiluminescence (PPLYGEN).

### Quantitative real-time PCR

Total RNA was isolated from frozen brain using TaKaRa MiniBEST Universal RNA Extraction Kit (TaKaRa, Kusatsu, Japan) according to the manufacturer's instructions. The total RNA (1 μg) was reverse transcribed to cDNA with PrimeScript™ RT reagent Kit with gDNA Eraser (TaKaRa, Kusatsu, Japan) according to the instructions manual. Quantitative real-time PCR was performed by using SYBR® Premix Ex Taq™ (TliRNaseH Plus) (TaKaRa, Kusatsu, Japan) combined with 10 ng of template cDNA and appropriate primers (Sangon Biotech, Shanghai, China) in a final volume of 20 μL. Data were collected and quantitatively analyzed using an ABI 7500 real-time PCR system (Applied Biosystems, Foster, CA, USA). PCR was performed as 95°C for 30 s, 40 cycles at 95°C for 5 s and 60°C for 30 s. Mouse β-actin mRNA level was used as an endogenous control for sample normalization. The relative mRNA expression was calculated as 2^−ΔΔ*CT*^. Sequences of PCR primers were as follows: for IFN-γ: forward, 5′-ATGAACGCTACACACTGCATC-3′, reverse, 5′-CCATCCTTTTGCCAGTTCCTC-3′, 182 bp; for IL-17A: forward, 5′-TCTCAGGCTCCCTCTTCAG-3′, reverse, 5′-GACTCTCCACCGCAATGA-3′, 161 bp; for T-bet: forward, 5′-ATTGGTTGGAGAGGAAGCGG-3′, reverse, 5′-GCACCAGGTTCGTGACTGTA-3′, 129 bp; for ROR-γt: forward, 5′-GGAACCAGAACAGGGTCCAG-3′, reverse, 5′-TAGAAGGTCCTCCAGTCGCA-3′, 154 bp; for β-actin: forward, 5′-ATATCGCTGCGCTGGTCGTC-3′, reverse, 5′-AGGATGGCGTGAGGGAGAGC-3′, 517 bp.

### Statistical analysis

Statistical analysis was performed with SPSS 18.0 software. The Student's *t*-test was used to analyze differences between two groups. Comparisons among the groups were made with ANOVA. All data were presented as mean ± SD. *P*-value < 0.05 was considered statistically significant.

## Results

### Characterization of PZH samples

HPLC study showed that the greatest absorbance wavelength was at 203 nm, and the HPLC analysis showed 7 characteristic peaks (Supplementary Figure 1). The chemical compositions of major peaks were analyzed and identified by HPLC-MS/TOF (Supplementary Figure 2, Supplementary Table 1) by comparison with the data of each reference substances (Notoginsenoside R1, Ginsenoside Rg1, Ginsenoside Rb1, Ginsenoside Re, Ginsenoside Rg2, Ginsenoside Rh1, Ginsenoside Rd). These characteristic patterns of HPLC chromatograms were used for standardization of the PZH samples in the subsequent studies.

### PZH effectively ameliorated the clinical symptoms of EAE mice

**Figure 1:**
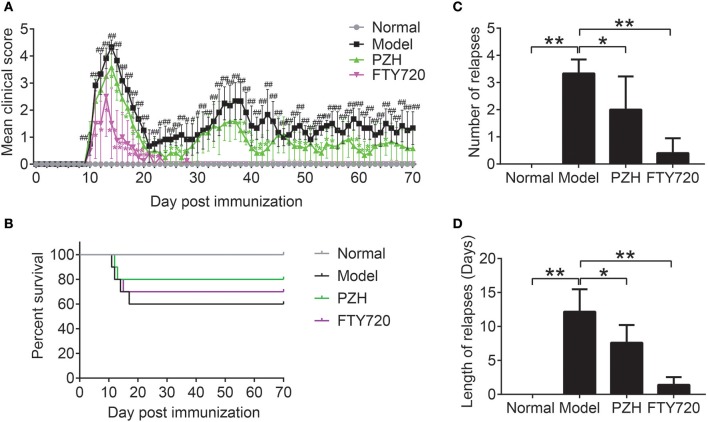
PZH ameliorated clinical symptoms of EAE mice. SJL mice were oral administered with distilled water or PZH (0.234 g/kg/d) or FTY720 (1 mg/kg/d) daily for 60 days from day 10 (disease onset) after immunization. **(A)** Time course changes of mean clinical score of mice from respective group. **(B)** The survival rate of mice in each group. **(C)** Number of relapse of mice in each group. Average number of relapses = the sum of the number of relapses of all mice in each group / the number of each group. **(D)** Length of relapses of mice in each group. Length of relapses (days) = the sum of the number of relapse days of all mice in each group / the number of each group. Results were shown as mean ± *SD* (*n* = 5 each group; ^#^*P* < 0.05, ^##^*P* < 0.01 when normal group vs. model group; ^*^P < 0.05, ^**^P < 0.01 when treatment group vs model group).

As shown in Figure 1A, no visible neurobehavioral deficits could be seen in normal mice over the entire period. Compared to the normal mice, EAE-associated clinical symptoms began to appear in model mice on the 10th day after immunization and the clinical scores of EAE mice rose quickly and reached a maximal level on day 14. The first phase of EAE alleviated with a low score on day 21 and spontaneously relapsed thereafter. The elevation of clinical scores were significantly inhibited both in FTY-720 group and PZH group from day 11 and day 14, respectively, with a complete suppression of EAE symptoms in FTY-720 group from day 28. In Figure 1B, it was shown that 40% mice died from EAE. PZH and FTY-720 treatment both inhibited the death rate of EAE mice. In addition, both FTY-720 and PZH could markedly reduce the number and length of relapses (Figures 1C,D).

### PZH ameliorated CNS inflammation and reduced demyelination

**Figure 2:**
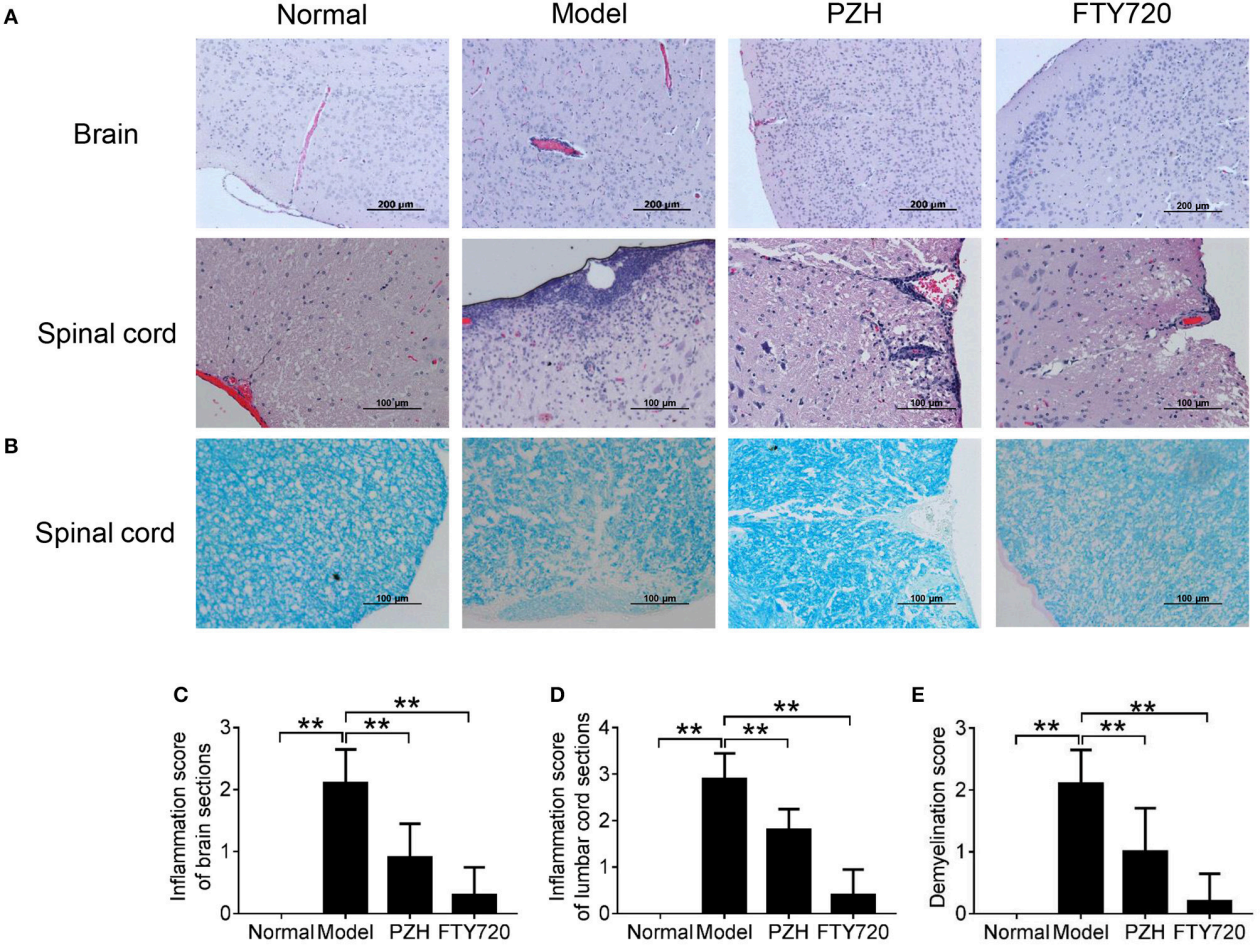
PZH ameliorated CNS inflammation and reduced demyelination in EAE mice. Mice were sacrificed at day 70 p.i. and the CNS were harvested. **(A)** Inflammation of brain and lumbar spinal cord was analyzed by H&E staining. **(B)** Demyelination of lumbar spinal cord was examined by Luxol fast blue (LFB) staining. **(C)** Inflammation score of the brain of mice in each group. **(D)** Inflammation score of the lumbar cord of mice in each group. **(E)** Demyelination score of the lumbar cord of mice in each group. Data were shown as mean ± *SD* (*n* = 5 in each group; ^**^P < 0.01).

To further investigate the effect of PZH on EAE mice, the inflammatory changes and the degree of demyelination in the CNS were observed by H&E staining and LFB staining, respectively. As shown in Figure 2A, model group showed apparent vascular cuff-like changes and diffused inflammatory cells infiltration in the brain and the lumbar cord compared with normal group. Both FTY-720 treatment and PZH treatment ameliorated the severity of these pathological changes. Moreover, LFB staining results demonstrated that FTY-720 and PZH could reduce the severity of demyelination in the lumbar cord of EAE mice (Figure 2B). Consistently, the inflammation scores and demyelination scores were significantly lower in the CNS of FTY-720-treated and PZH-treated mice than that of untreated EAE mice (Figures 2C–E).

### PZH reduced pro-inflammatory cytokines expression in EAE mice

**Figure 3:**
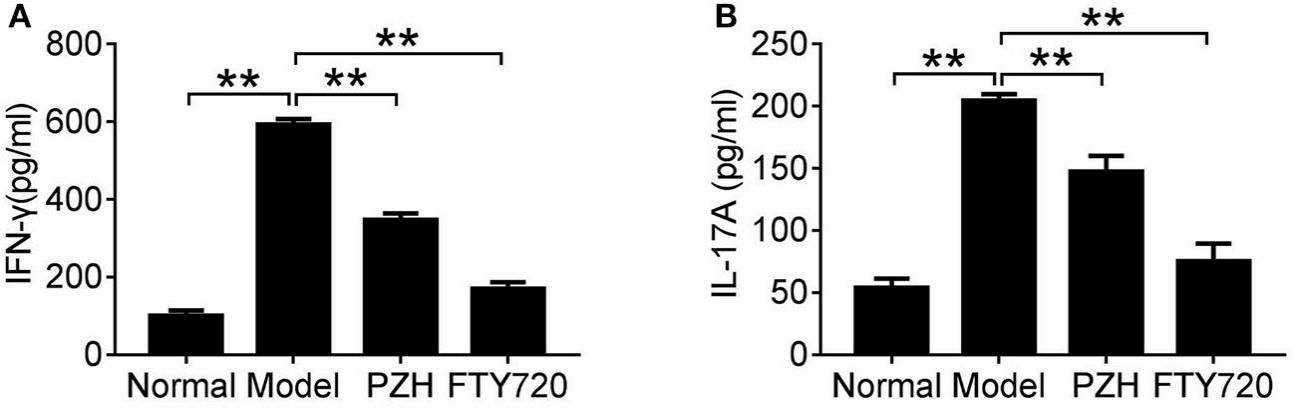
PZH reduced pro-inflammatory cytokines expression in serum of EAE mice. Mice were sacrificed at day 70 p.i. and the serum was collected for ELISA detection. The levels of IFN-γ **(A)** and IL-17A **(B)** were shown respectively. Data were shown as mean ± *SD* (*n* = 5 in each group; ^**^P < 0.01).

To investigate whether PZH played an anti-inflammatory effect in EAE mice, we detected the levels of two important pro-inflammatory cytokines in sera of EAE mice. As shown in Figure 3, compared to normal group, the levels of IFN-γ and IL-17A were significantly increased in model group, whereas the elevated cytokines were remarkably decreased in both PZH group and FTY-720 group.

### PZH lowered the percentage of Th1 and Th17 cell subsets

**Figure 4:**
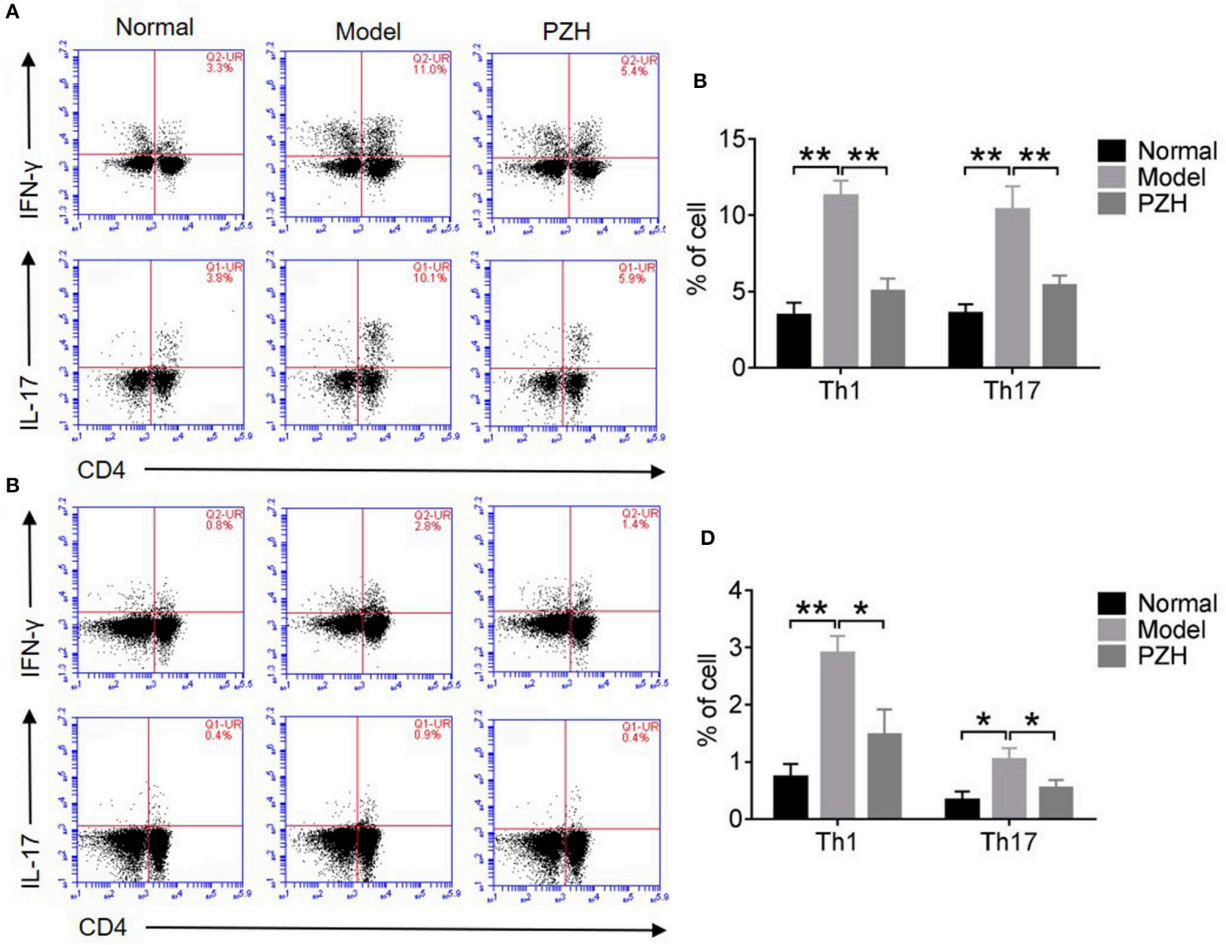
PZH reduced the percentages of Th1 and Th17 cell subsets. Mice were sacrificed at day 70 p.i., and the MNCs in the CNS and spleen were harvested. **(A)** Subsets of Th1 and Th17 cells in CD4+ gate were analyzed by intracellular staining of IFN-γ and IL-17 respectively for MNCs in the CNS. **(B)** Percentages of Th1 cells and Th17 cells in the CNS of mice from each group. **(C)** Subsets of Th1 and Th17 cells in CD4+ gate were analyzed by intracellular staining of IFN-γ and IL-17 respectively for MNCs in spleen. **(D)** Percentages of Th1 cells and Th17 cells in the spleen of mice from each group. All data were expressed as mean ± *SD* (*n* = 5 each group; ^*^P < 0.05; ^**^P < 0.01).

As shown in Figures 4A–D, both Th1 and Th17 cell subsets in the CNS and spleen of mice from model group were remarkably increased compared to that from normal group. PZH treatment significantly reduced the percentages of Th1 and Th17 cell subsets both in the CNS and the spleen of EAE mice.

### PZH inhibited the activities of Th1 and Th17 cell subsets

**Figure 5:**
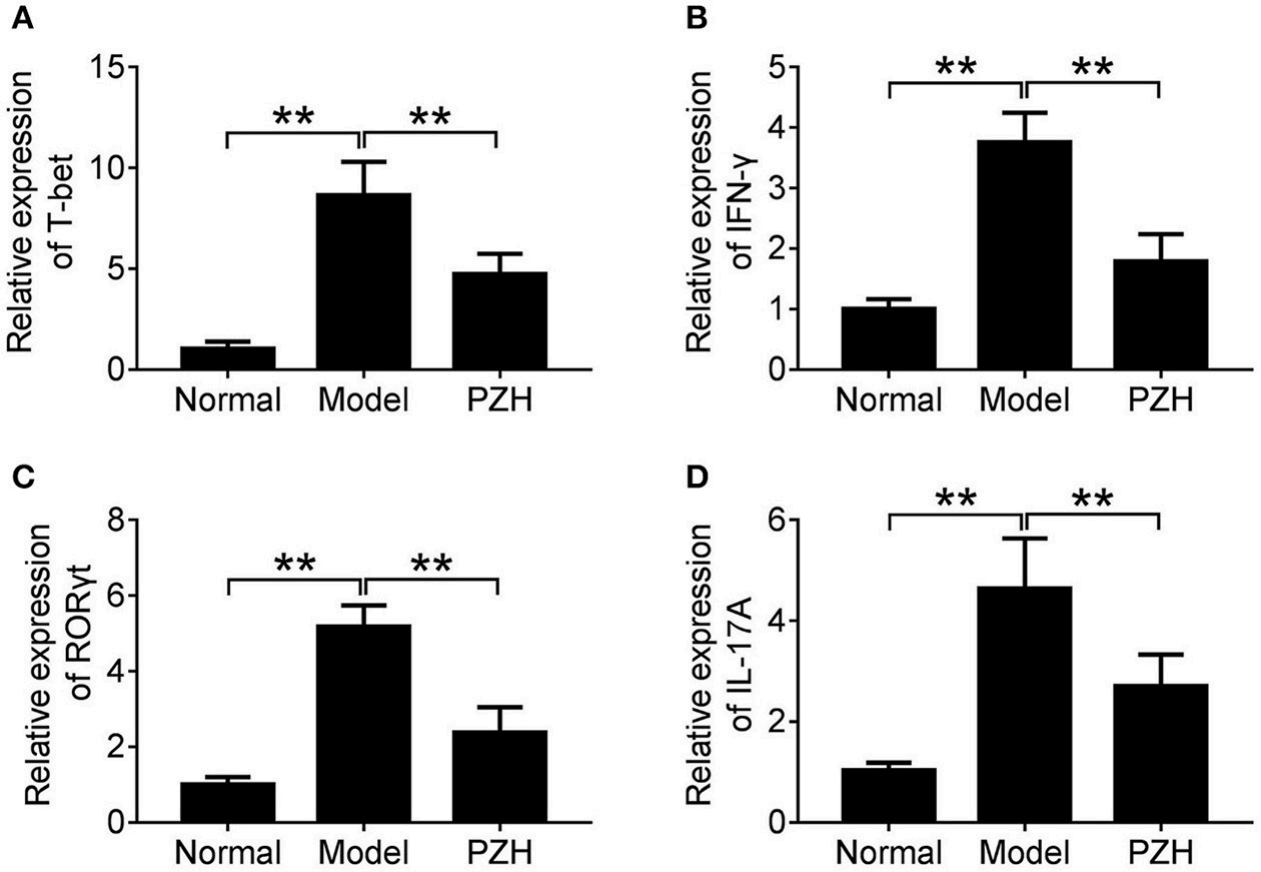
PZH inhibited the activities of Th1 and Th17 cell subsets. Mice were sacrificed at day 70 p.i., and the brain tissue were collected. The mRNA levels of T-bet **(A)**, IFN-γ **(B)**, ROR-γt **(C)**, and IL-17A **(D)** were analyzed by real-time PCR. All data were expressed as mean ± *SD* (*n* = 5 each group; ^*^P < 0.05; ^**^P < 0.01).

To further investigate whether PZH could regulate the activities of Th1 and Th17 cells, we detected the mRNA levels of T-bet and ROR-γt, two key transcription factors for Th1 and Th17 cells respectively, as well as that of pro-inflammatory cytokines IFN-γ and IL-17A in the brain of mice. The results showed that PZH significantly down-regulated the mRNA levels of T-bet and IFN-γ which were related with Th1 cells (Figures 5A,B). Similarly, the mRNA levels of ROR-γt for Th17 cells and the downstream pro-inflammatory cytokine IL-17A was also decreased in PZH group compared with model group (Figures 5C,D).

### PZH modulated the STAT and NF-κB signaling pathways

**Figure 6:**
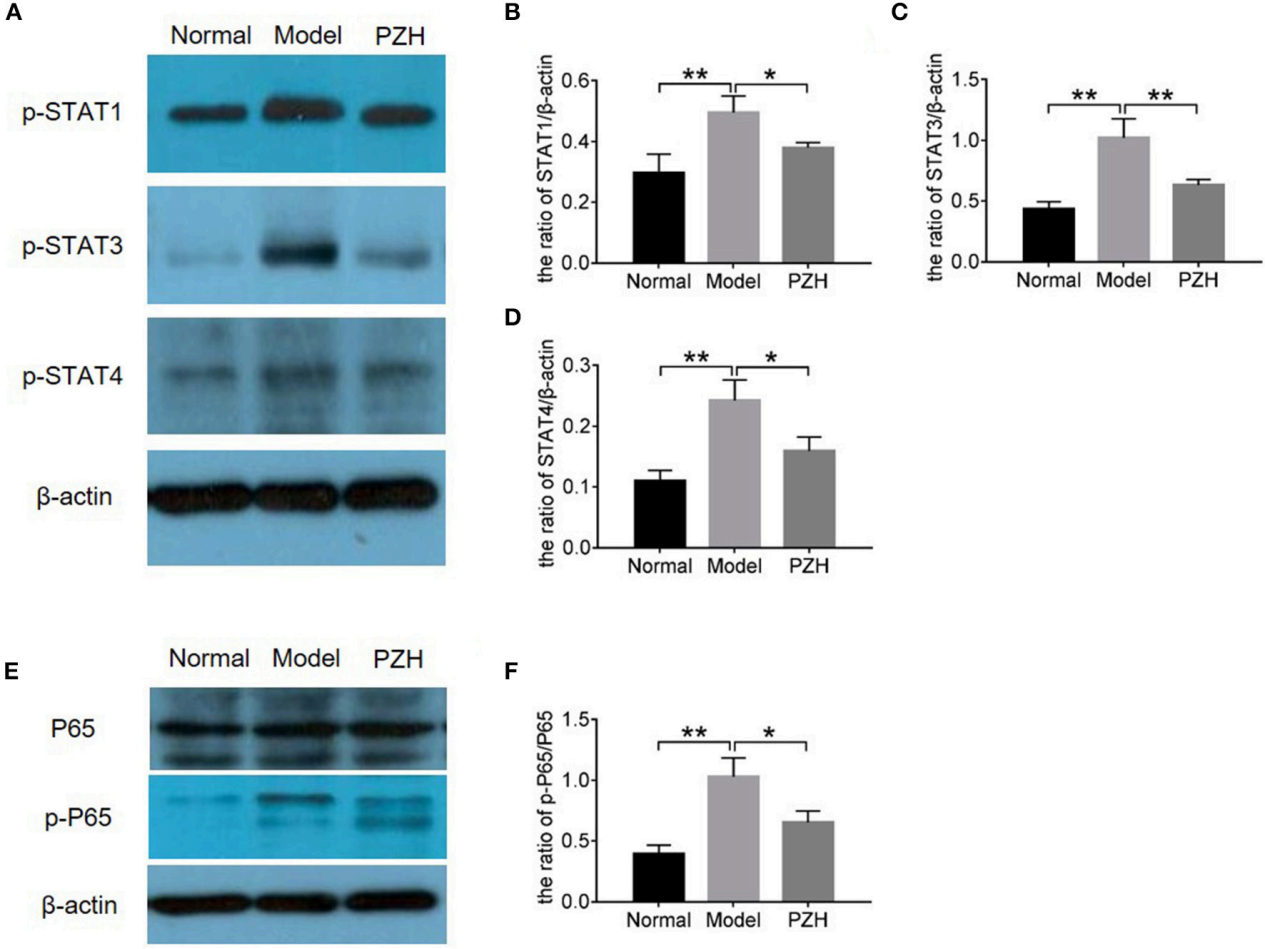
PZH regulated the STAT and NFκB signaling pathways. Mice were sacrificed at day 70 p.i., and the brain tissue were used for western blot detection. **(A)** The levels of p-STAT1, p-STAT3, and p-STAT4 were detected by western blot. **(B–D)** The quantitative analysis results of p-STAT1, p-STAT3, and p-STAT4. **(E)** The levels of P65 and p-P65 were detected by western blot. **(F)** The ratio of p-P65/P65. All data were expressed as mean ± *SD* (*n* = 5 each group; ^*^P < 0.05; ^**^P < 0.01).

In order to further study the mechanism of PZH inhibiting Th1 and Th17 differentiation and activities, proteins in STAT and NF-κB signaling pathways were measured by western blot. Here, the results demonstrated that the levels of p-STAT1 and p-STAT4 for Th1 cells as well as p-STAT3 for Th17 cells were significantly decreased in the brain of EAE mice after PZH treatment (Figures 6A–D), proving the regulation effect of PZH on STAT signaling pathway. Moreover, the decreased ratio of p-P65/P65 in PZH-treated mice compared to model mice (Figures 6E–F) indicated that PZH also modulated NF-κB signaling pathway.

## Discussion

RR-EAE in SJL mice immunized with PLP_139−151_/CFA is an universal mouse model which most closely resembles human remitting-relapsing MS (Furlan et al., 2009; Xie et al., 2016). The typical EAE clinical signs is weakness or paralysis of the tail (score 1), followed by a progression up the body to affect the hind limbs (score 3) and finally the forelimbs (score 4) (de Bruin et al., 2016). According to our data, PZH treatment remarkably attenuated the clinical symptoms of EAE mice, not only in onset phase but also in relapse phase, and had significant effect on reducing the frequency of relapse. Furthermore, to fully evaluate the efficacy of PZH to the lesion of CNS, we observed the pathological changes in the brain and lumbar cord of mice. It can be found that the typical pathological changes of vascular cuffing infiltration of inflammatory cells in the brain and lumbar cord of EAE mice remarkably alleviated by PZH treatment. These results further confirmed the therapeutic effect of PZH on EAE mice.

The pivotal roles of Th1 and Th17 cells in the pathogenesis of MS and EAE have been proved by numerous studies (Zhang et al., 2014; Yadav et al., 2015). Th1 cells primarily produce IFN-γ, which acts as the most potent macrophage activator in the cellular immunity. And there is intact importance of Th1 cells in the development of MS that is characterized by predominantly mononuclear cell infiltration (Zhang et al., 2014). Myelin-specific Th17 cells traffic into the CNS, where they secrete IL-17A, which through chemokine induction attracts various immune cells, and in particular myeloid cells, into the CNS, initiating and perpetuating the inflammatory cascade (Rostami and Ciric, 2013). Like disease initiation, relapses are characterized by aggregated Th1/Th17 immune responses (Steinman, 2014; Yadav et al., 2015). Targeting Th1 or Th17 cells could also reduce relapse number or relapse rate (Farjam et al., 2015; Balasa et al., 2017). Here, the study firstly observed that the sera levels of IFN-γ and IL-17A in PZH-treated EAE mice were significantly lower than that in untreated EAE mice. Then it was found that PZH treatment significantly decreased the percentages of Th1 and Th17 cells in both spleen and CNS of EAE mice. T-bet and ROR-γt are key transcription factors for Th1 and Th17 cells respectively. The expression levels of these transcription factors are associated with the release of proinflammatory cytokines, including IFN-γ, IL-17A, and so on (Chen et al., 2016; Feng et al., 2016). T-bet is also necessary for EAE development, as T-bet^−/−^ mice are resistant to EAE (Bettelli et al., 2004). Continuous RORγt expression is required to maintain the functions of Th17 cells *in vivo* (Bettelli et al., 2008). Down-regulation of ROR-γt suppressed Th17 differentiation and effector function (Nosratabadi et al., 2016). Targeting RORγt in Th17 cells or T-bet in Th1 cells could be therapeutically beneficial in the treatment of inflammatory autoimmune diseases (Garber, 2011; Park et al., 2014). It is worth noting that our results showed that PZH decreased the mRNA levels of T-bet and RORγt in the brain of EAE mice. It also inhibited the mRNA expression of IFN-γ and IL-17A in EAE mice brain. All results implicated that PZH could modulate the differentiation and function of Th1 and Th17 cell subsets in RR-EAE mice which may account for the beneficial effect of PZH on relapses of RR-EAE.

Previous studies found that the differentiation of T cell subsets was regulated by STAT pathway, the low level of p-STAT1 and p-STAT4 for Th1 cells and p-STAT3 for Th17 cells leading to reduction in numbers of Th1 and Th17 cells (Zhang et al., 2015). Moreover, mice deficient in STAT4 are resistant to the induction of EAE, with minimal inflammatory infiltrates in the CNS (Chitnis et al., 2001). Previous studies showed that PZH could suppress STAT3 signaling (Shen et al., 2012; Zhuang et al., 2012). In the present study, we found that PZH effectively decreased the levels of p-STAT1, p-STAT3, and p-STAT4 in the brain of EAE mice. On the other hand, aberrant NF-κB activation contributes to the development of MS. Through a cascade of phosphorylation, the kinase complex is activated and NF-κB enter the nucleus to upregulate genes involved in T-cell proliferation, maturation and development (Park and Hong, 2016). Our study demonstrated that PZH had limited effect on the expression of P65 but decreased the production of p-P65, which inhibited the activation of NF-κB pathway.

## Conclusion

This study demonstrated that PZH had a therapeutic effect on EAE mice, which was associated with inhibiting the differentiation and function of Th1 and Th17 cells.

## Author contributions

XQ, QG, and XL performed the major research in equal contribution. HL, DF, YD, HC, CL, and GZ provided the technical support. XH designed the study and revised the manuscript. AL contributed to final approval of the version to be published.

### Conflict of interest statement

The authors declare that the research was conducted in the absence of any commercial or financial relationships that could be construed as a potential conflict of interest.
