# Supplementary material for: Pien Tze Huang Alleviates Relapsing-Remitting Experimental Autoimmune Encephalomyelitis Mice by Regulating Th1 and Th17 Cells
Source: Front Pharmacol. 2018 Oct 31;9:1237. doi: 10.3389/fphar.2018.01237 (PMC6220046; doi:10.3389/fphar.2018.01237)
Supplement: Supplementary file 1 [file Data_Sheet_1.PDF]

Supplementary Material

Pien Tze Huang Alleviates Relapsing-remitting Experimental Autoimmune Encephalomyelitis Mice by Regulating Th1 and Th17 Cells

Xuemei Qiu[1,2+], Qingqing Guo[1,3+], Xue Liu[1,2+], Hui Luo[1,2], Danping Fan[1], Yongqi Deng[1,2], Hua Cui[1,2], Cheng Lu[1], Ge Zhang[3], Xiaojuan He[1,3]* and Aiping Lu[3,4]*

*Correspondence: Xiaojuan He, hxj19@126.com; Aiping Lu, aipinglu@hkbu.edu.hk

† These authors contributed equally to this work.

1 Supplementary Tables and Figures

1.1 Supplementary Figures

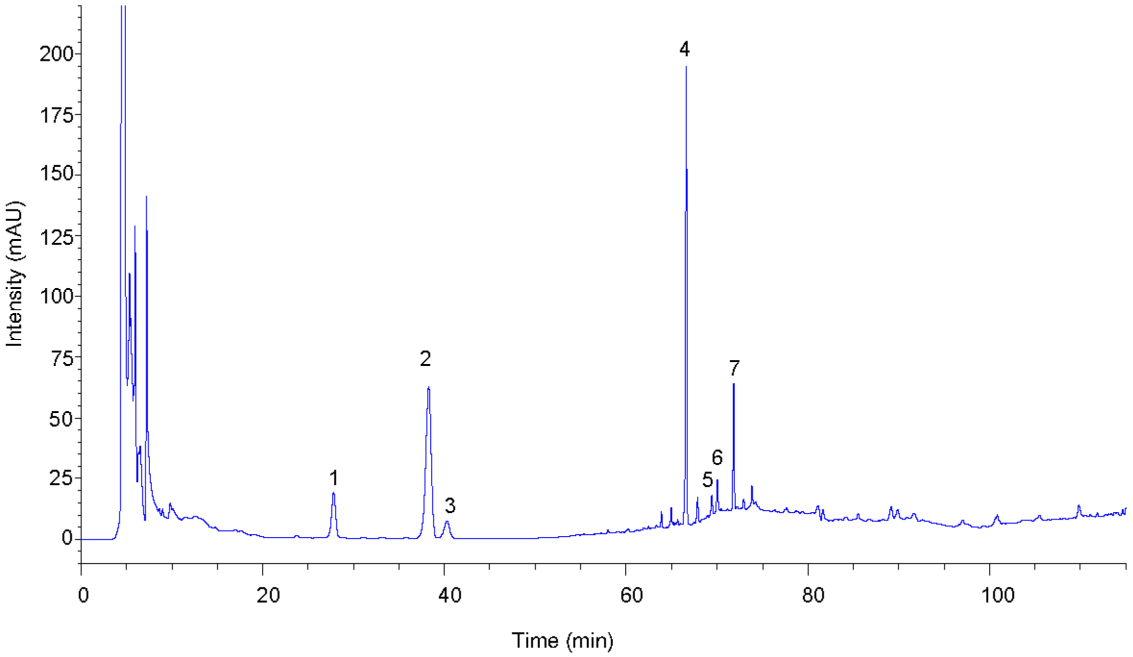

Supplementary Figure 1. HPLC-finger print of PTH measured at 203 nm.

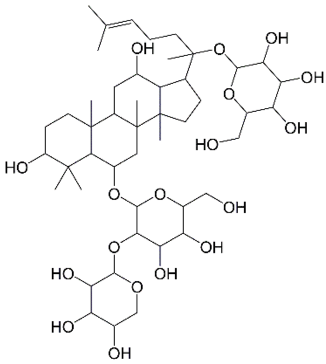

Notoginsenoside R1

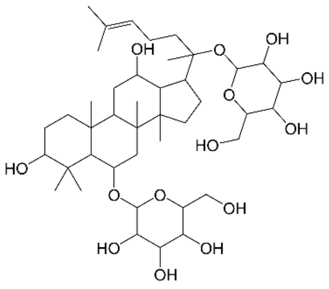

Ginsenoside Rg1

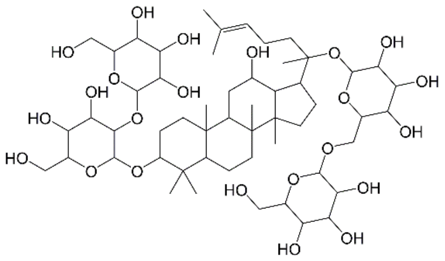

Ginsenoside Rb1

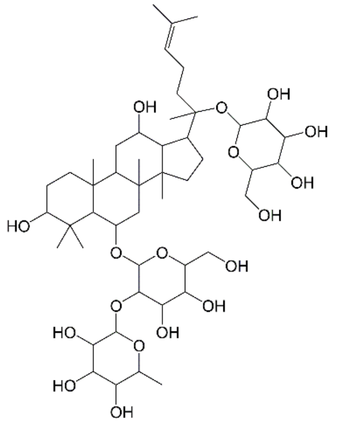

Ginsenoside Re

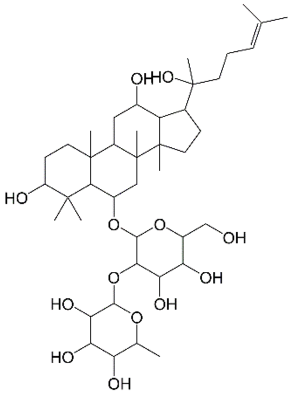

Ginsenoside Rg2

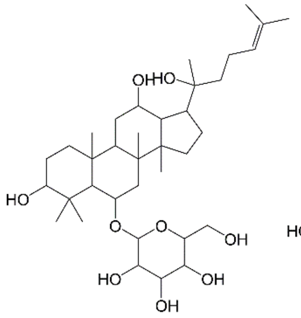

Ginsenoside Rh1

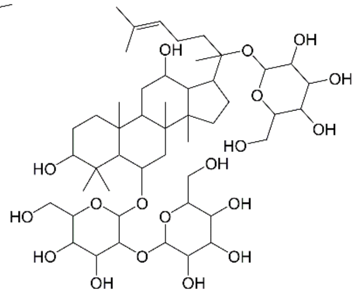

Ginsenoside Rd

Supplementary Figure 2. Chemical structures of 7 components in PZH.

1.2 Supplementary Tables

Supplementary Table 1. Components identified in PZH

Peak	Compounds	Retention time	Molecular formula	Negative mode	CAS number	Source
1	Notoginsenoside R1	23.15	C47H80O18	$[M-H]^-$=932.1393	80418-24-2	PN
2	Ginsenoside Rg1	39.11	C42H72O14	$[M-H]^-$=799.4836	22427-39-0	PN
3	Ginsenoside Rb1	40.76	C54H92O23	$[M-H]^-$=1107.5959	41753-43-9	PN
4	Ginsenoside Re	66.85	C48H82O18	$[M-H]^-$=945.5435	52286-59-6	PN
5	Ginsenoside Rg2	69.13	C42H72O13	$[M-H]^-$=783.4891	52286-74-5	PN
6	Ginsenoside Rh1	70.64	C36H62O9	$[M-H]^-$=637.4312	63223-86-9	PN
7	Ginsenoside Rd	72.45	C48H82O18	$[M-H]^-$= 945.5423	52705-93-8	PN
